# The importance of dopamine levels and single-nucleotide polymorphism within *COMT*, *DRD1* and *DRD2* genes in obstructive sleep apnoea

**DOI:** 10.1080/07853890.2025.2548386

**Published:** 2025-08-20

**Authors:** Joanna Smardz, Helena Martynowicz, Marta Dratwa-Kuzmin, Anna Wojakowska, Pawel Gac, Katarzyna Bogunia-Kubik, Mieszko Wieckiewicz

**Affiliations:** a Department of Experimental Dentistry, Faculty of Dentistry, Wroclaw Medical University, Wroclaw, Poland; b Clinical Department of Diabetology, Hypertension and Internal Diseases, Institute of Internal Diseases, Faculty of Medicine, Wroclaw Medical University, Wroclaw, Poland; c Laboratory of Clinical Immunogenetics and Pharmacogenetics, Hirszfeld Institute of Immunology and Experimental Therapy, Polish Academy of Sciences, Wroclaw, Poland; d Department of Environmental Health, Occupational Medicine and Epidemiology, Faculty of Health Sciences, Wroclaw Medical University, Wroclaw, Poland

**Keywords:** Obstructive sleep apnoea, dopamine, COMT, DRD1, DRD2, genetics, neurotransmission

## Abstract

**Background:**

Obstructive sleep apnoea (OSA) is a prevalent sleep disorder that contributes to serious cardiovascular comorbidities. While the mechanical aspects of OSA are well-studied, its neurobiological underpinnings remain underexplored. In this study, we investigated the role of dopamine and its genetic modulators in OSA pathophysiology.

**Patients and methods:**

Serum dopamine levels were assessed in a cohort of 153 participants (96 OSA patients and 57 controls), and single-nucleotide polymorphisms (SNPs) in dopamine-related genes, including *COMT*, *DRD1* and *DRD2*, were analysed in a cohort of 286 participants (141 OSA patients and 145 controls).

**Results:**

Elevated serum dopamine levels were observed in OSA patients (*p* = 0.01), with dopamine levels correlating independently with OSA and male gender. Genotypic analysis identified the *DRD2* rs1800497 *T* allele as a potential independent predictor of OSA severity (*p* = 0.011), hypopnea (*p* = 0.005) and arousals (*p* = 0.024).

**Conclusions:**

This study advances the understanding of OSA by identifying elevated dopamine levels and genetic variations in *DRD2* rs1800497 as potential modulators of its occurrence and severity. These findings pave the way for personalized diagnostic and therapeutic approaches. By integrating neurobiology, genetics, and clinical practice, this research contributes to the evolving framework for precision medicine in sleep disorders.

## KEY MESSAGES

- Patients with obstructive sleep apnoea (OSA) were observed to have elevated serum dopamine levels.
- Genotypic analysis identified the DRD2 rs1800497 T allele as a potential independent predictor of OSA severity.

## 1. Introduction

Obstructive sleep apnoea (OSA) is a prevalent sleep disorder characterized by repetitive episodes of partial or complete upper airway obstruction during sleep [1]. These episodes lead to intermittent hypoxia, hypercapnia and arousal, which disrupt normal sleep architecture [1,2]. The condition results in excessive daytime sleepiness, and reduced quality of life, and is associated with significant comorbidities. It is a risk factor for hypertension, poorly controlled blood pressure, stroke, myocardial infarction, heart failure, cardiac arrhythmias, sudden cardiac death and all-cause mortality [1–3]. Approximately 1 billion of the world’s population of 7.3 billion people, between the ages of 30 and 69 years, are estimated to have OSA – the most common type of sleep-disordered breathing [4]. Despite advances in understanding the mechanical aspects of OSA, the neurobiological and genetic underpinnings of the condition remain underexplored.

Dopamine, chemically known as 4-(2-aminoethyl) benzene-1,2-diol, is a catecholamine neurotransmitter synthesized primarily in the central nervous system and adrenal medulla [5]. Dopamine plays a crucial role in regulating arousal, respiratory drive, and upper airway muscle tone through its action on dopamine receptors (D1–D5), which are G-protein-coupled receptors distributed across various brain regions and peripheral tissues [6]. Alterations in dopaminergic signalling have been implicated in conditions such as restless legs syndrome and narcolepsy, suggesting a broader role for dopamine in sleep-related disorders [6–9]. Moreover, dopamine has emerged as a potential modulator in the pathogenesis of OSA [10–13]. Genetic variants within dopamine pathway genes, including catechol-O-methyltransferase (*COMT*) and dopamine receptor genes (*DRD1* and *DRD2*), have been associated with neurobehavioral traits and sleep behaviours, such as sleep bruxism, which commonly co-occurs with OSA. However, their specific contribution to OSA pathophysiology remains poorly defined [14–16].

Recent advances in genomics and statistical modelling provide a unique opportunity to dissect the complex interactions between genetic predisposition, dopamine signalling and environmental factors contributing to OSA. Understanding these interactions could pave the way for novel diagnostic markers and targeted therapies, significantly improving healthcare for OSA patients and mitigating the health, social and economic consequences associated with the disorder [1–4,10–17].

The aim of this study was to: (a) assess dopamine blood levels and (b) evaluate four single-nucleotide polymorphisms (SNPs) within the *COMT*, *DRD1* and *DRD2* genes in a cohort of OSA participants and healthy controls. The selected SNPs may have functional implications and play regulatory roles. The *COMT* rs4680 and rs6269 SNPs have been implicated in functional connectivity, cognitive function, sleep and bruxism [18–21]. The *DRD1* rs686 SNP has been reported to influence microRNA interactions and potentially affect predisposition to sleep bruxism [14,22]. The *DRD2* rs1800497 SNP has been associated with Parkinson’s disease, mood disorders, sleep dysfunction and sleep bruxism [15,23,24]. By employing a multidisciplinary approach integrating biochemical assays, genomic analyses and comprehensive statistical modelling, this study aims to elucidate the neurogenetic mechanisms underlying OSA. Addressing these gaps could advance the understanding of OSA pathophysiology and identify avenues for precision medicine in the management of this pervasive sleep disorder.

## 2. Patients and methods

Information on research methods has been partially described in our previous study [25].

### 2.1. Participants

Participants were recruited in 2023 and 2024 from adults hospitalized at the Department and Clinic of Internal Medicine, Occupational Diseases, Hypertension and Clinical Oncology, Wroclaw Medical University. The study adhered to the guidelines of the Declaration of Helsinki and was approved by the Ethical Committee of Wroclaw Medical University (ID: 199/2023). Written informed consent was obtained from all participants. Information about clinical trial registration is available at www.ClinicalTrials.gov (identifier: NCT05890911). The study was funded by Wroclaw Medical University (grant number SUBK.B160.23.059).

### 2.2. Polysomnography

NoxA1 system (NOX Medical, Reykjavík, Iceland) was used to conduct audio-video polysomnography (avPSG) in the Sleep Laboratory of the Department and Clinic of Internal Medicine, Occupational Diseases, Hypertension and Clinical Oncology, Wroclaw Medical University. Recordings were performed from 10:00 pm to 6:00 am. Standard elements assessed during avPSG included electroencephalographic, electrocardiographic, electrooculographic and electromyographic recordings from the chin area and bilaterally from the masseter muscles, as well as recordings of abdominal and thoracic breathing activity, body position and audio–video recordings. NONIN WristOx2 3150 pulse oximeter (Nonin Medical Inc., Plymouth, MN) was used to record pulse, saturation level and plethysmographic data. Noxturnal software (Nox Medical, Reykjavík, Iceland) was used to reconstruct recordings. All avPSG recordings were scored and analysed in 30-s epochs by a qualified and experienced physician by the American Academy of Sleep Medicine (AASM) *Manual for the Scoring of Sleep and Associated Events* version 2.4 [26,27].

The parameters measured included the apnoea/hypopnea index (AHI), oxygen desaturation index (ODI), apnoea index (AI), obstructive apnoea (OA), central apnoea (CA), mixed apnoea (MI), hypopnea index (HI), average and minimal saturation < 90% and arousals [26,27].

Based on the 2013 AASM standard criteria for sleep scoring [27], comparative analyses showing the lack, the presence and the severity of OSA were carried out in the following groups:

1. AHI < 5 and AHI ≥ 5,
2. AHI < 15 and AHI ≥ 15,
3. AHI < 30 and AHI ≥ 30,
4. ODI < 5 and ODI ≥ 5 and
5. average saturation < 93% and average saturation ≥ 93%.

According to the AASM, AHI was categorized into mild (5–15 events/h), moderate (15–30 events/h) and severe (> 30 events/h) [27].

### 2.3. Inclusion and exclusion criteria

The inclusion criteria for the study were age ≥18 years and willingness to participate. Exclusion criteria included severe systemic disorders and diseases (including genetic disorders, as per existing hospital protocols); neurological disorders and/or neuropathic pain; active inflammation; active malignancy; severe mental disorders or significant mental (including genetic) disabilities; pregnancy or confinement; treatment with or addiction to analgesic agents or drugs affecting the nervous system, muscles, respiratory system or sleep; and refusal to participate.

Participants diagnosed with OSA through avPSG (AHI ≥5) were included in the study group, while those without OSA (AHI <5) were included in the control group.

### 2.4. Assessment of blood dopamine level

Peripheral blood samples (4 mL) were collected from each participant between 8:00 and 9:00 am the morning following avPSG, using the Vacutainer^®^ blood sampling system (Becton Dickinson, Franklin Lakes, NJ). Samples were collected into anticoagulant-free tubes and centrifuged at 4500 × *g* for 10 min to separate the serum. The serum was transferred to 1.5-mL Eppendorf tubes (Eppendorf, Hamburg, Germany), frozen, and stored at −80 °C until analysis.

Serum dopamine levels were measured using a commercially available enzyme-linked immunosorbent assay (ELISA) kit (ab285238, Abcam, Cambridge, UK) with a sensitivity of <0.938 ng/mL. The assay was based on the principles of competitive ELISA and was conducted according to the manufacturer’s protocol, using the overnight incubation version. Optical density (OD) was measured with a Sunrise microplate reader and Magellan analysis software (Tecan Trading AG, Männedorf, Switzerland) at 405 nm, with a reference wavelength of 600 nm. Dopamine concentrations were quantified, and each sample was measured in duplicate. The mean serum dopamine levels (ng/mL) were calculated for each participant based on a standard curve fit.

### 2.5. DNA isolation and genotyping

Peripheral blood samples (10 mL) were collected from each participant between 8:00 and 9:00 am the morning following avPSG, using the Vacutainer^®^ blood sampling system (Becton Dickinson, Franklin Lakes, NJ). Samples were collected into ethylenediaminetetraacetic acid (EDTA)-containing tubes and frozen at −20 °C until further use. DNA was extracted using the NucleoSpin Blood L Kit (Macherey-Nagel, Düren, Germany), following the manufacturer’s instructions. DNA concentration and purity were measured using a DeNovix DS-11 spectrophotometer (DeNovix Inc., Wilmington, DE). The extracted DNA was stored at −20 °C until SNP genotyping.

The genotyping of *COMT* rs4680 and rs6269, *DRD1* rs686 and *DRD2* rs1800497 polymorphisms was performed using the LightSNiP typing assay (TIB-MolBiol, Berlin, Germany), which involved amplification by quantitative polymerase chain reaction (qPCR) and subsequent melting curve analysis. Reactions were conducted on a LightCycler 480 II system (Roche Diagnostics, Rotkreuz, Switzerland), adhering to the manufacturer’s protocol.

The reaction programme included an initial denaturation step at 95 °C for 10 min, followed by 45 cycles of denaturation at 95 °C for 10 s, annealing at 60 °C for 10 s and extension at 72 °C for 15 s. Melting curve analysis followed, starting with a 30-s incubation at 95 °C, then 2 min at 40 °C, with continuous data acquisition during the melting process between 40 and 75 °C at a ramp rate of 1.5 °C/s.

### 2.6. Statistical analysis

Data analysis was conducted using TIBCO Software Inc.’s Statistica software version 13 (http://statistica.io). Results were considered statistically significant at *p* < 0.05. Data distribution and deviations from normality were assessed using the Shapiro–Wilk test, with *p* < 0.05 indicating non-normal distribution. Between-group differences were analysed using the Mann–Whitney *U* test, while correlation analysis was performed using Spearman’s rank correlation test. Multivariate regression analysis was also applied. Dopamine concentration or quantitative polysomnographic variables were assessed as dependent variables in various regression models. Anthropometric parameters (quantitative age and dichotomous sex) and the presence of alleles at the studied gene locus (as dichotomous variables) were included as potential variables independently associated with the dependent variables in these models. The regression models were estimated using the least squares method.

Sample size was calculated using a sample size calculator. The parameters included a population size of 3 million (the population of the Lower Silesian Voivodeship, Poland), a fraction size of 0.5 (the estimated percentage of people with OSA in this population), a maximum error of 10% (a restrictive threshold for this type of research) and a confidence level of 95% (standard statistical significance). The required minimum sample size was determined to be 96 participants. For the dopamine level assessment (153 participants), the maximum error was estimated at 8% and for SNP evaluation (286 participants), it was estimated at 6%.

## 3. Results

### 3.1. Dopamine levels

#### 3.1.1. Comparative analyses

**Figure 1.**
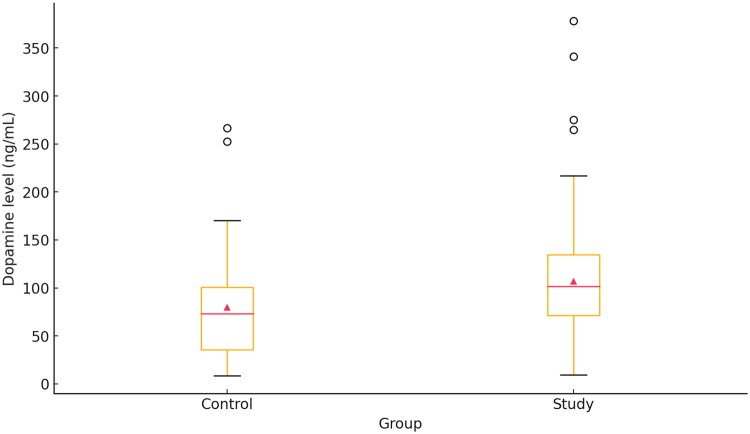
Comparison of dopamine levels between groups.

**Figure 2.**
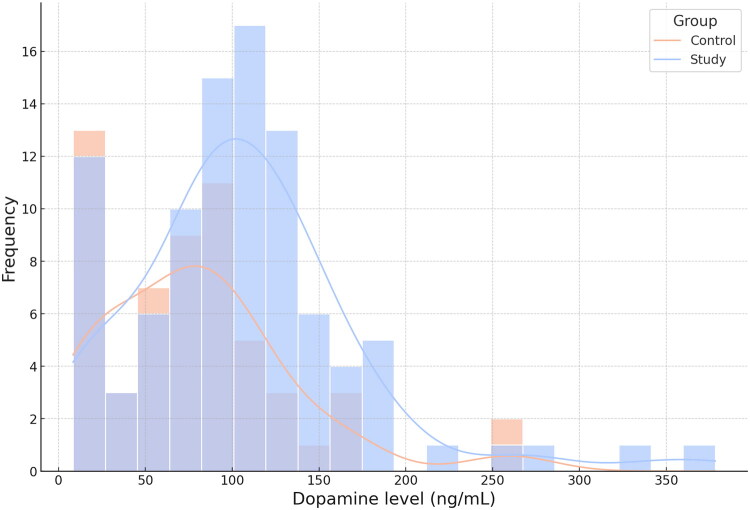
Distribution of dopamine levels by group.

The study group consisted of 96 Caucasian patients (44 females and 52 males; mean age: 52.69 ± 13.99 years; age range: 20–79 years) diagnosed with OSA through avPSG. The control group included 57 Caucasian participants (33 females and 24 males; mean age: 38.53 ± 11.62 years; age range: 21–68 years) without OSA, confirmed *via* avPSG. Statistical analysis revealed that blood dopamine levels were significantly higher in the study group compared to the control group (*p* = 0.01) (Table 1, Figures 1 and 2). Table 1 provides descriptive statistics for the studied variables in the study and control groups.

**Table 1.** Descriptive statistics for study and control groups in dopamine levels analyses.

Variable	Study group (*n* = 96)				Control group (*n* = 57)			
	Average	Minimum	Maximum	SD	Average	Minimum	Maximum	SD
Age (years)	52.69	20.00	79.00	13.99	38.53	21.00	68.00	11.62
Dopamine level (ng/mL)*	106.88	9.42	378.17	64.85	80.10	8.39	266.66	54.88
AHI (/h)	23.72	5.20	87.40	20.02	2.15	0.10	4.90	1.43
ODI (/h)	22.72	1.30	88.40	19.27	2.62	0.00	7.10	1.70
Snore (%)	30.15	0.00	87.90	21.22	8.42	0.00	63.10	14.24
AI (/h)	9.62	0.00	72.40	15.45	0.66	0.00	3.10	0.69
OA (/h)	8.44	0.00	71.20	14.27	0.31	0.00	1.70	0.40
MA (/h)	0.38	0.00	11.70	1.48	0.02	0.00	0.30	0.06
CA (/h)	0.72	0.00	10.90	1.66	0.32	0.00	2.60	0.54
HI (/h)	14.09	0.80	54.20	10.16	1.49	0.00	4.20	1.12
Average saturation (%)	92.02	74.60	95.80	3.05	94.54	84.70	97.00	2.39
Minimal saturation (%)	81.32	51.00	95.00	8.15	87.75	52.00	94.00	6.75
Saturation <90% (%)	14.48	0.00	86.60	20.66	1.65	0.00	31.00	5.25
Arousals (/h)	7.91	0.00	88.30	13.38	3.43	0.00	10.60	2.12

AHI: apnoea/hypopnea index; ODI: oxygen desaturation index; AI: apnoea index; OA: obstructive apnoea; MA: mixed apnoea, CA: central apnoea, HI: hypopnea index; (/h): per hour; SD: standard deviation; *n*: number of participants

* *p* = 0.01 for comparison of dopamine levels between the study and control group.

Comparative analyses in groups: (1) AHI < 5 *vs.* AHI ≥ 5, (2) AHI < 15 *vs*. AHI ≥ 15; (3) AHI < 30 *vs*. AHI ≥ 30; (4) ODI < 5 *vs.* ODI ≥ 5; (5) average saturation < 93% *vs*. average saturation ≥ 93%; and average saturation < 90% *vs.* average saturation ≥ 90% showed statistically significant differences in analyses 1 and 4 (*p* = 0.01 and *p* = 0.008; respectively). Participants with AHI and ODI lower than 5 had significantly lower blood dopamine levels (Table 2).

**Table 2.** Comparative analyses of dopamine levels.

Comparison	Assumption (1)		Assumption (2)		*p* Value
	Average dopamine level (ng/mL)	SD	Average dopamine level (ng/mL)	SD	
AHI < 5 (1) *vs*. AHI ≥ 5 (2)	80.1	54.88	106.88	64.85	0.01
AHI < 15 (1) *vs*. AHI ≥ 15 (2)	92.72	62.61	104.36	62.17	0.27
AHI < 30 (1) *vs*. AHI ≥ 30 (2)	96.02	62.66	101.45	62.78	0.692
ODI < 5 (1) *vs.* ODI ≥ 5 (2)	78.65	58.89	106.81	62.47	0.008
Average saturation < 93% (1) *vs.* average saturation ≥ 93% (2)	99.34	56.76	95.01	66.90	0.672
Average saturation < 90% (1) *vs.* average saturation ≥ 90% (2)	93.26	44.9	97.27	64.12	0.820

(1): assumption (1); (2): assumption (2); AHI: apnoea/hypopnea index; ODI: oxygen desaturation index; SD: standard deviation

#### 3.1.2. Correlation analyses

In analyses performed for merged dopamine levels study and control group (*n* = 153), dopamine levels were not significantly correlated with parameters, such as AHI, ODI, CA, OA, MA, HI, average and minimal saturation, saturation <90% or arousals (*p* > 0.05 for all comparisons).

#### 3.1.3. Multivariate regression analysis

Multivariate regression analysis performed for merged dopamine levels study and control group (*n* = 153) identified OSA and male gender as independent risk factors for higher dopamine levels (*p* = 0.005 and *p* = 0.007, respectively). The analysis did not show any statistically significant correlations for age (*p* = 0.074).

### 3.2. Evaluation of SNPs

#### 3.2.1. Comparison analyses

The study group for SNP evaluation consisted of 141 Caucasian patients (60 females and 81 males; mean age: 51.39 ± 14.14 years; age range: 20–79 years) diagnosed with OSA *via* avPSG. The control group comprised 145 Caucasian participants (102 females and 43 males; mean age: 34.59 ± 10.75 years; age range: 18–68 years) without OSA, confirmed through avPSG.

Table 3 presents the descriptive statistics for the studied variables in the study and control groups.

**Table 3.** Descriptive statistics for study and control groups in single-nucleotide polymorphisms (SNPs) analyses.

Variable	Study group (*n* = 141)				Control group (*n* = 145)			
	Average	Minimum	Maximum	SD	Average	Minimum	Maximum	SD
Age (years)	51.39	20.00	79.00	14.14	34.59	18.00	68.00	10.75
AHI (/h)	23.71	5.00	87.40	19.48	1.94	0.00	4.90	1.35
ODI (/h)	22.36	0.80	88.40	18.98	2.34	0.00	14.20	1.83
Snore (%)	29.37	0.00	87.90	21.81	6.00	0.00	63.10	11.14
AI (/h)	9.61	0.00	77.50	15.31	0.49	0.00	3.10	0.61
OA (/h)	8.10	0.00	71.20	13.59	0.20	0.00	1.80	0.36
MA (/h)	0.54	0.00	25.20	2.49	0.01	0.00	0.30	0.05
CA (/h)	0.74	0.00	10.90	1.56	0.27	0.00	2.60	0.44
HI (/h)	14.09	0.10	54.20	10.17	1.46	0.00	4.70	1.11
Average saturation (%)	92.27	74.60	96.30	2.78	94.95	84.70	97.30	1.81
Minimal saturation (%)	81.57	51.00	95.00	8.19	88.91	52.00	95.00	6.74
Saturation <90% (%)	12.93	0.00	86.60	19.75	1.14	0.00	31.00	3.93
Arousals (/h)	7.39	0.00	88.30	11.85	4.00	0.00	31.70	3.47

AHI: apnoea/hypopnea index; ODI: oxygen desaturation index; AI: apnoea index; OA: obstructive apnoea; MA: mixed apnoea; CA: central apnoea; HI: hypopnea index; (/h): per hour; SD: standard deviation; *n:* number of participants

**Figure 3.**
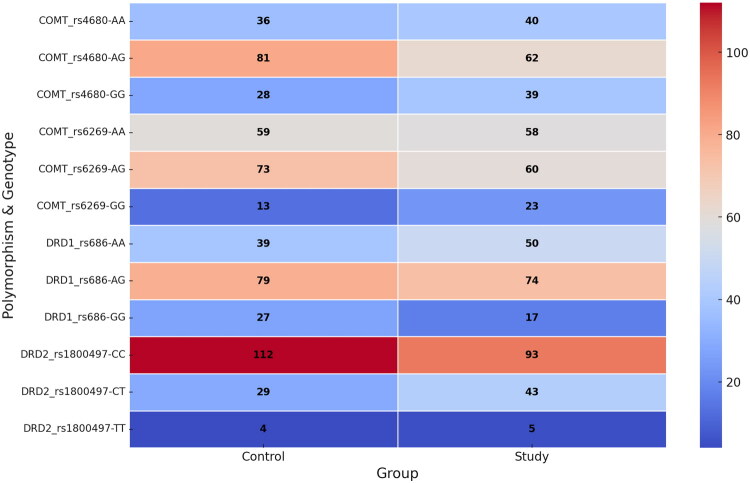
Heatmap of genotype distribution by group.

Comparison of SNP genotypes between the study and control groups revealed no significant differences (*p* > 0.05 for all comparisons). The genotype distribution for *COMT* rs4680 and rs6269, *DRD1* rs686 and *DRD2* rs1800497 in both groups is shown in Table 4 and Figure 3.

**Table 4.** Distribution of the *COMT* (rs4680 and rs6269), *DRD1* (rs686) and *DRD2* (rs1800497) genotypes in the study and control group.

Polymorphism	Study group, *n* (%)	Control group, *n* (%)	*p* Value
*COMT* rs4680			
*AA*	40 (28.37)	36 (24.83)	ns
*AG*	62 (43.97)	81 (55.86)	ns
*GG*	39 (27.66)	28 (19.31)	ns
*COMT* rs6269			
*AA*	58 (41.13)	59 (40.69)	ns
*AG*	60 (42.55)	73 (50.34)	ns
*GG*	23 (16.31)	13 (8.97)	ns
*DRD1* rs686			
*AA*	50 (35.46)	39 (26.9)	ns
*AG*	74 (52.48)	79 (54.48)	ns
*GG*	17 (12.06)	27 (18.62)	ns
*DRD2* rs1800497			
*CC*	93 (65.96)	112 (77.24)	ns
*CT*	43 (30.5)	29 (20)	ns
*TT*	5 (3.55)	4 (2.76)	ns

*n:* number of participants with a given genotype; ns: nonsignificant

Statistical analysis of alleles in the studied loci showed that the *T* allele at the *DRD2* rs1800497 locus occurred more frequently in the study group (*p* = 0.036), while the *A* allele at the *COMT* rs6269 locus occurred less frequently (*p* = 0.041). Significant correlations between individual alleles and variables assessed during avPSG are summarized in Table 5.

**Table 5.** Statistically significant findings regarding the correlation of the presence of individual alleles within the studied genes loci with the variables examined during polysomnography.

Variable	Allele present average ± SD	Allele absent average ± SD	*p* Value
*COMT* rs4680 allele *A*			
Minimal saturation (%)	85.84 ± 7.7	83.52 ± 9.98	0.047
Saturation < 90% (%)	5.76 ± 12.67	10.85 ± 21.46	0.017
*COMT* rs4680 allele *G*			
OA (/h)	3.36 ± 9.95	6.19 ± 11.14	0.042
*COMT* rs6269 allele *A*			
ODI (/h)	11.40 ± 15.37	17.86 ± 23.54	0.03
Average saturation (%)	93.75 ± 2.4	92.76 ± 4.16	0.038
Saturation < 90% (%)	6.05 ± 13.3	13.19 ± 24.54	0.009
*DRD1* rs686 allele *A*			
Minimal saturation (%)	84.86 ± 8.64	87.7 ± 5.86	0.037
*DRD1* rs686 allele *G*			
AHI (/h)	11.23 ± 15.91	15.86 ± 20.32	0.038
ODI (/h)	10.9 ± 15	15.11 ± 19.76	0.048
HI (/h)	6.82 ± 8.23	9.59 ± 11.83	0.023
Average saturation (%)	93.88 ± 2.34	93.07 ± 3.3	0.018
*DRD2* rs1800497 allele *T*			
AHI (/h)	17.37 ± 20.79	10.82 ± 15.69	0.004
ODI (/h)	17.02 ± 20.19	10.31 ± 14.74	0.002
HI (/h)	10.80 ± 11.42	6.45 ± 8.44	0.000
Arousals (/h)	7.57 ± 11.99	4.84 ± 6.86	0.018

AHI: apnoea/hypopnea index; ODI: oxygen desaturation index; OA: obstructive apnoea; HI: hypopnea index; (/h): per hour; SD: standard deviation

#### 3.2.2. Multivariate regression analysis

Multivariate regression analysis performed for merged SNPs study and control group (*n* = 286) identified male gender, older age and the presence of the *T* allele at the *DRD2* rs1800497 locus as independent predictors of higher AHI (*p* = 0.000, *p* = 0.000 and *p* = 0.011, respectively), ODI (*p* = 0.000, *p* = 0.000 and *p* = 0.005, respectively) and HI (*p* = 0.003, *p* = 0.000 and *p* = 0.001, respectively). Additionally, male gender and older age were independent predictors of higher OA (*p* = 0.000 and *p* = 0.000, respectively) and lower average saturation (*p* = 0.005 and *p* = 0.000, respectively) while the presence of the *G* allele at the *COMT* rs4680 locus was a protective factor against higher OA (*p* = 0.023). Furthermore, the *T* allele at the *DRD2* rs1800497 locus was an independent predictor of a higher arousal rate (*p* = 0.024).

## 4. Discussion

This study investigates the intersection of dopamine dysregulation, genetic predispositions and the pathophysiology of OSA. By integrating biochemical, genetic and clinical analyses, it provides novel insights into potential biomarkers and therapeutic targets, while emphasizing the need for further investigation to unravel the complexities of OSA.

The significantly elevated dopamine levels observed in OSA patients highlight the potential role of neurotransmitter imbalance in disease pathophysiology. Dopamine’s involvement in regulating arousal, respiratory drive and muscle tone supports its hypothesized role as a compensatory mechanism in response to intermittent hypoxia and disrupted sleep architecture [10,11]. Conversely, chronic hypoxia may dysregulate dopamine metabolism, contributing to elevated levels. This dual role suggests that dopamine may serve as both a marker and a modulator of OSA [11,28–30]. Previous studies have linked elevated dopamine levels to disrupted sleep patterns and neural compensation mechanisms, reinforcing its potential significance in OSA [6,29,30]. Moreover, extraneuronal dopamine elevation, observed in rats exposed to moderate hypoxia [31], has been implicated in neuronal destruction. However, it remains unclear whether dopamine elevation is a precursor to OSA or a secondary effect of its pathophysiological processes. The absence of significant correlations between dopamine levels and OSA severity markers (AHI, ODI) observed in this study may suggest that dopamine elevation represents a compensatory mechanism rather than a direct severity indicator. The observed dopamine elevation may reflect a complex interplay between direct respiratory control mechanisms and systemic inflammatory/oxidative stress responses characteristic of OSA [32].

The genetic analysis adds further depth to the understanding of dopamine-related pathways in OSA. The *DRD2* rs1800497 *T* allele emerged as potential independent predictor of OSA occurrence, including higher arousal rates and AHI, aligning with previous research linking this polymorphism to sleep behaviours and neuropsychiatric conditions [23,24]. Notably, the *G* allele of the *DRD2* rs1800497 SNP has been associated with a significant reduction in the risk of awake-sleep bruxism [15], a phenomenon hypothesized to act as a protective mechanism against hypoxia in OSA. Although other SNPs studied did not show significant differences between groups, their established associations with cognitive, movement and sleep functions in prior studies [14,18,19] remain relevant. These findings suggest that specific dopamine-related polymorphisms may influence OSA severity rather than its occurrence, underscoring the complex genetic landscape of OSA. This highlights the need to explore the polygenic nature of OSA, where multiple genetic and environmental factors interact to shape disease expression.

The combination of elevated dopamine levels and potential predictive role of the *DRD2* rs1800497 polymorphism underscores the importance of dopamine signalling in OSA pathophysiology. However, the lack of significant findings for other SNPs points to the necessity of studying polygenic contributions, including the interplay of multiple genetic variants influencing dopamine pathways in OSA [4,14,17]. Additionally, gene–environment interactions should be explored to understand how factors, such as obesity, stress and physical activity modulate genetic predispositions [18,23, 33,34]. Future research could leverage integrative genomic and transcriptomic approaches to unravel these intricate relationships [10,19]. While our findings suggest a direct role of dopamine in OSA pathophysiology, we acknowledge that indirect mechanisms through oxidative stress, inflammation and metabolic dysfunction may also contribute to the observed associations. The observed dopamine elevation may reflect a complex interplay between direct respiratory control mechanisms and systemic inflammatory/oxidative stress responses characteristic of OSA [32].

**Figure 4.**
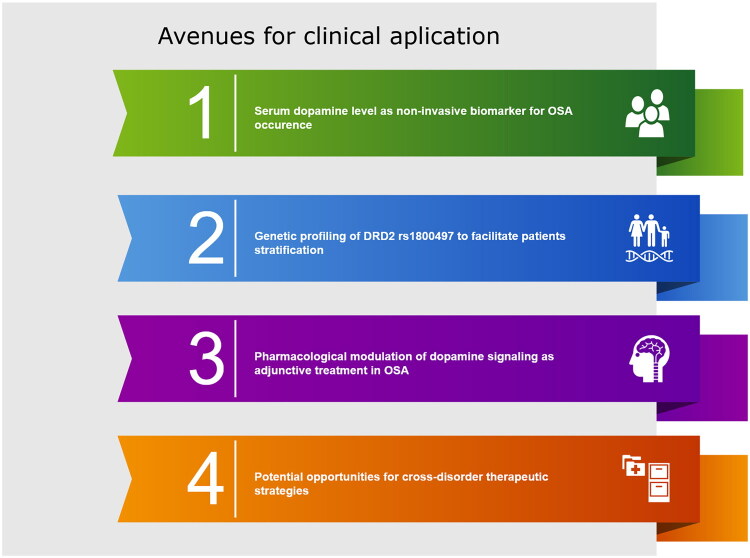
Avenues of clinical application.

The study provides several potential avenues for clinical application. The significant elevation of dopamine in OSA patients suggests its potential as a noninvasive biomarker for disease occurrence, complementing traditional diagnostic tools [4]. However, while our findings demonstrate elevated dopamine in OSA patients, the absence of direct correlations with traditional severity metrics suggests complexity in its potential biomarker utility. Dopamine elevation may reflect compensatory neuroadaptation rather than disease severity, limiting its use as a standalone diagnostic tool. Similar to other proposed OSA biomarkers (e.g. inflammatory markers) [32], dopamine shows promise for understanding disease mechanisms but requires integration with clinical parameters for practical utility. Genetic profiling of dopamine-related SNPs, such as *DRD2* rs1800497, could facilitate patient stratification based on risk and inform tailored treatment strategies [10]. Pharmacological modulation of dopamine signalling may offer a novel therapeutic approach; for instance, dopamine receptor agonists or antagonists could be explored as adjunctive treatments, particularly in severe OSA cases [6,35–38]. However, these interventions require careful consideration of potential side effects, such as mood or motor disturbances [8]. The relevance of dopamine signalling extends beyond OSA to other sleep and neuropsychiatric disorders, such as restless legs syndrome, narcolepsy and Parkinson’s disease. Shared mechanisms, such as hypoxia-induced dopamine dysregulation, suggest opportunities for cross-disorder therapeutic strategies [7, 8]. Dopamine dysregulation’s role in motor control, arousal and respiratory function underscores its broader significance in overlapping conditions [6,10,39]. Therapeutic approaches targeting dopamine pathways in one disorder may have translational potential across multiple disorders involving dopaminergic dysfunction [4]. The summary of potential avenues for clinical application is presented in Figure 4.

This study demonstrates several significant strengths, contributing to a deeper understanding of OSA and its neurogenetic mechanisms. By integrating biochemical, genetic and clinical methodologies, the study provides a comprehensive examination of dopamine signalling’s role in OSA. This multidisciplinary framework ensures a holistic perspective, addressing an underexplored aspect of the disorder. The evaluation of blood dopamine levels and dopamine-related genetic polymorphisms offers valuable insights into the neurochemical basis of OSA. The inclusion of avPSG ensures precise and standardized diagnosis and classification of OSA severity [40–42], enhancing the reliability of clinical data.

The genetic analysis focuses on SNPs in dopamine pathway genes, such as *DRD2* rs1800497 and *COMT* rs4680, potentially linking genetic variations to OSA occurrence and severity. This adds a novel dimension to OSA research, with implications for personalized medicine. Elevated dopamine levels are proposed as a noninvasive biomarker for OSA severity, potentially offering practical applications for improving diagnosis and disease monitoring. The use of robust statistical methods, including regression analyses, strengthens the identification of independent predictors and associations, bolstering the study’s conclusions. Moreover, by investigating correlations between dopamine, genetic polymorphisms and OSA severity rather than mere presence, the study provides clinically relevant insights for risk stratification and treatment strategies.

The study’s relatively large sample size, compared to other research in related fields, enhances its robustness and generalizability. Collectively, these strengths establish the study as a valuable contribution to sleep medicine, laying a strong foundation for future research and clinical applications.

However, several limitations highlight areas for future improvement. The study’s focus on a Caucasian population restricts the generalizability of its findings, as genetic risk factors may vary across ethnic groups, necessitating multiethnic cohorts for broader applicability [17]. The study and control groups are not perfectly matched in terms of age and gender due to limitations in access to polysomnography dependent on the conditions of the Polish national healthcare system. The study does not include experiments to validate how SNPs influence dopamine receptor expression or function, leaving the mechanistic implications unaddressed [23]. Also, while our findings suggest a direct role of dopamine in OSA pathophysiology more, we acknowledge that indirect mechanisms through oxidative stress, inflammation and metabolic dysfunction may also contribute to the observed associations. Lack of data on body mass index, levels of inflammatory markers, sleep quality, psychoemotional disturbances, comorbidities and OSA treatment may be also considered limitations of this study.

Future research directions include conducting genome-wide association studies across diverse populations to identify novel risk loci, investigating epigenetic modifications, such as DNA methylation, that could influence dopamine-related genes in OSA [19], and integrating genomic data with proteomic and metabolomic analyses to explore the functional consequences of identified polymorphisms [24].

## 5. Conclusion

This study advances the understanding of OSA by identifying elevated dopamine levels and genetic variations in *DRD2* rs1800497 as potential modulators of its occurrence and severity. These findings pave the way for personalized diagnostic and therapeutic approaches. Although our findings suggest dopamine dysregulation in OSA, it requires validation in larger, age-matched cohorts before clinical implementation. Future research should focus on dopamine’s role in OSA phenotyping rather than severity assessment. Addressing the study’s limitations and expanding the scope of future research will be essential to fully realize the clinical potential of these discoveries. By integrating neurobiology, genetics, and clinical practice, this research contributes to the evolving framework for precision medicine in sleep disorders.

## Data Availability

General data are provided with this article. All the other data are available from the corresponding author (M.W.), upon reasonable request because of the European Union General Data Protection Regulation (GDPR).
